# Calcific tendinitis of the rotator cuff: state of the art in diagnosis and treatment

**DOI:** 10.1007/s10195-015-0367-6

**Published:** 2015-07-12

**Authors:** Giovanni Merolla, Sanjay Singh, Paolo Paladini, Giuseppe Porcellini

**Affiliations:** Unit of Shoulder and Elbow Surgery, D. Cervesi Hospital, Cattolica (RN) - AUSL della Romagna Ambito Territoriale di Rimini, Italy; Biomechanics Laboratory “Marco Simoncelli”, D. Cervesi Hospital, Cattolica (RN) - AUSL della Romagna Ambito Territoriale di Rimini, Italy

**Keywords:** Calcific tendinitis, Shoulder, Rotator cuff, Diagnosis, Treatment options

## Abstract

Calcific tendinitis is a painful shoulder disorder characterised by either single or multiple deposits in the rotator cuff tendon. Although the disease subsides spontaneously in most cases, a subpopulation of patients continue to complain of pain and shoulder dysfunction and the deposits do not show any signs of resolution. Although several treatment options have been proposed, clinical results are controversial and often the indication for a given therapy remains a matter of clinician choice. Herein, we report on the current state of the art in the pathogenesis, diagnosis and treatment of calcific tendinitis of the rotator cuff.

## Introduction

Calcific tendinitis (CT) is a painful shoulder disorder characterised by either single or multiple deposits in the rotator cuff (RC) tendon or subacromial bursa [1]. It was Codman who, in his book [2], described the deposits as being in the RC tendon. The term “calcifying tendinitis” was probably first coined by Plenk [3] in 1952. The disease subsides spontaneously in the majority of cases and can be managed with conservative therapy, but some patients continue to have a painful shoulder for an extended period of time with the deposits not showing any signs of resolution. New conservative treatment modalities such as ultrasound-guided needling (UGN) and extracorporeal shock wave therapy (ESWT) have emerged in recent years as additional management options. Incidence varies from 2.7 to 20 %, as reported by various authors [1, 4, 5]. In about 10–20 % of patients, the deposits are bilateral [1, 5, 6]. Most studies found higher incidence in women compared with men [1, 6]. Regarding age distribution, the average age of presentation in most studies was between 30 and 50 years [5, 6]. No deposits were found in the elderly [5, 7, 8]. Most investigators found the deposits to be more commonly located in the supraspinatus [1, 3, 4, 6], although often the deposits were also located in the infraspinatus [1, 4, 6] and rarely in the subscapularis and teres minor [1, 4]. Most patients were sedentary workers or housewives [6]. The right shoulder was most commonly affected [6]. The natural history of the disease can be divided into three distinct clinical stages: acute, subacute and chronic. The main clinical manifestation is pain, which may or may not be associated with acute or gradual restriction of movements [4, 9]. Acute pain is often associated with the onset of the disease; however, the deposits may be asymptomatic in 20 % of cases [6]. Muscle spasm, and inflammation of subacromial bursa (bursitis) and the long head of the biceps are determining symptomatic factors. The pain is, in most cases, associated with the acute phase of the disease, but episodes of acute pain are also often related to flare-ups of chronic tendinopathy or onset of rare complications not related to the evolution of the disease, such as adhesive capsulitis (AC), rotator cuff tear, pathology of the long head of the biceps or osteolysis of the greater tuberosity (TO) [10, 11].

**Fig. 1:**
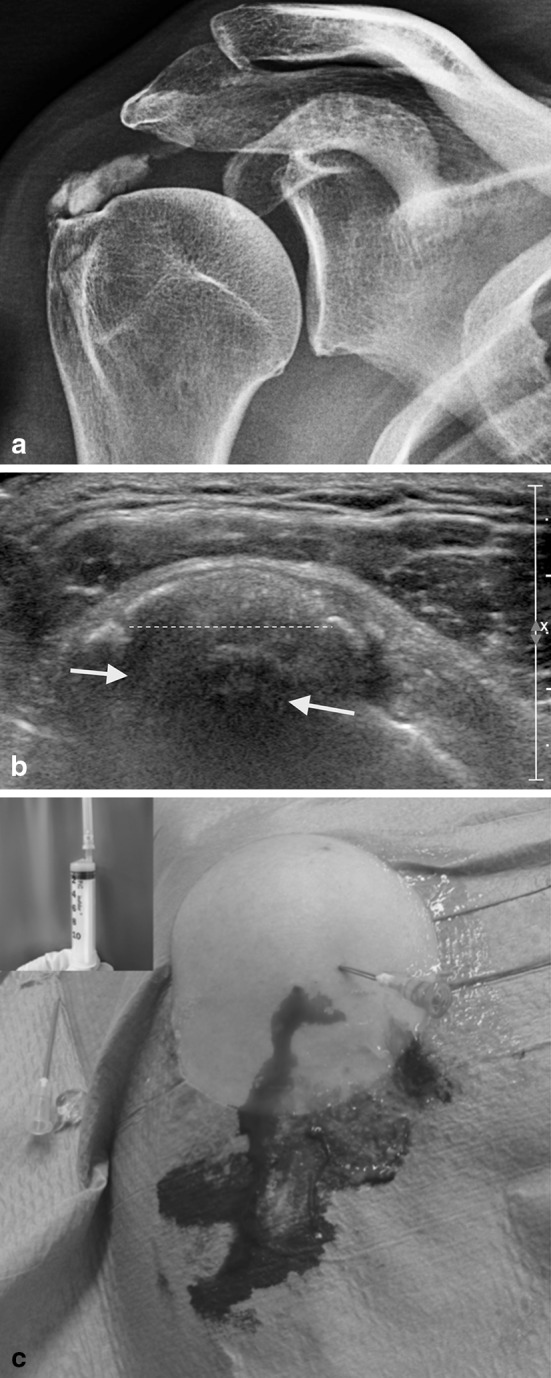
A case with acute calcifying tendinitis of the rotator cuff. (**a**) X-ray shows a large calcium deposit (>1.5 cm) at the insertion of the supraspinatus tendon in touch with the greater tuberosity; (**b**) ultrasound image in the same patient as **a** demonstrates a large fragmented and punctate calcification (*dotted line*) with hypoechoic area indicating oedema associated with the reabsorptive phase (*white arrows*); (**c**) ultrasound-guided needling and lavage in the same case as** a** and **b** with an abundant leakage of calcium (the window on the *left* shows the calcium aspirated in a syringe)

**Fig. 2:**
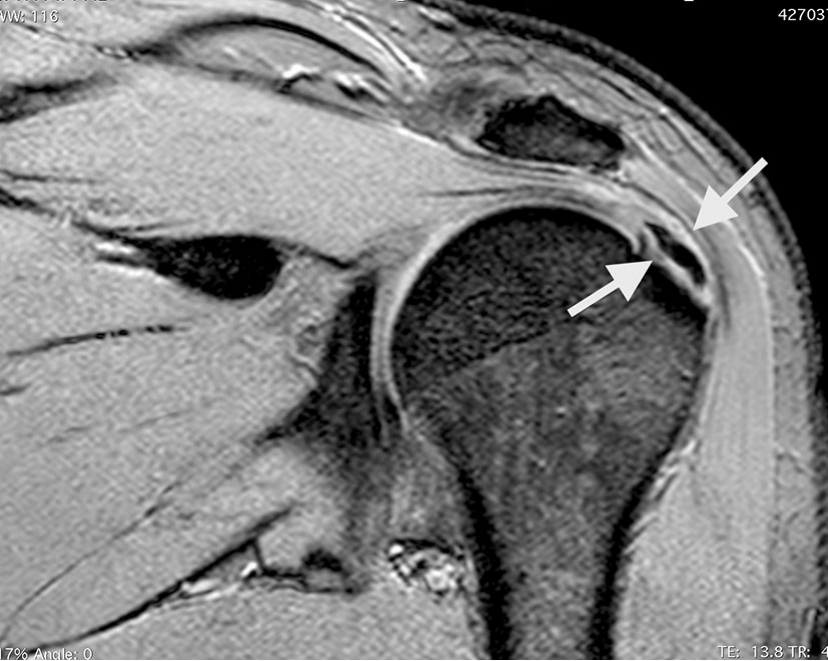
Coronal fatty suppressed MRI reveals a focus of chronic calcification with associated full-thickness supraspinatus tendon tear (*white arrows*)

**Fig. 3:**
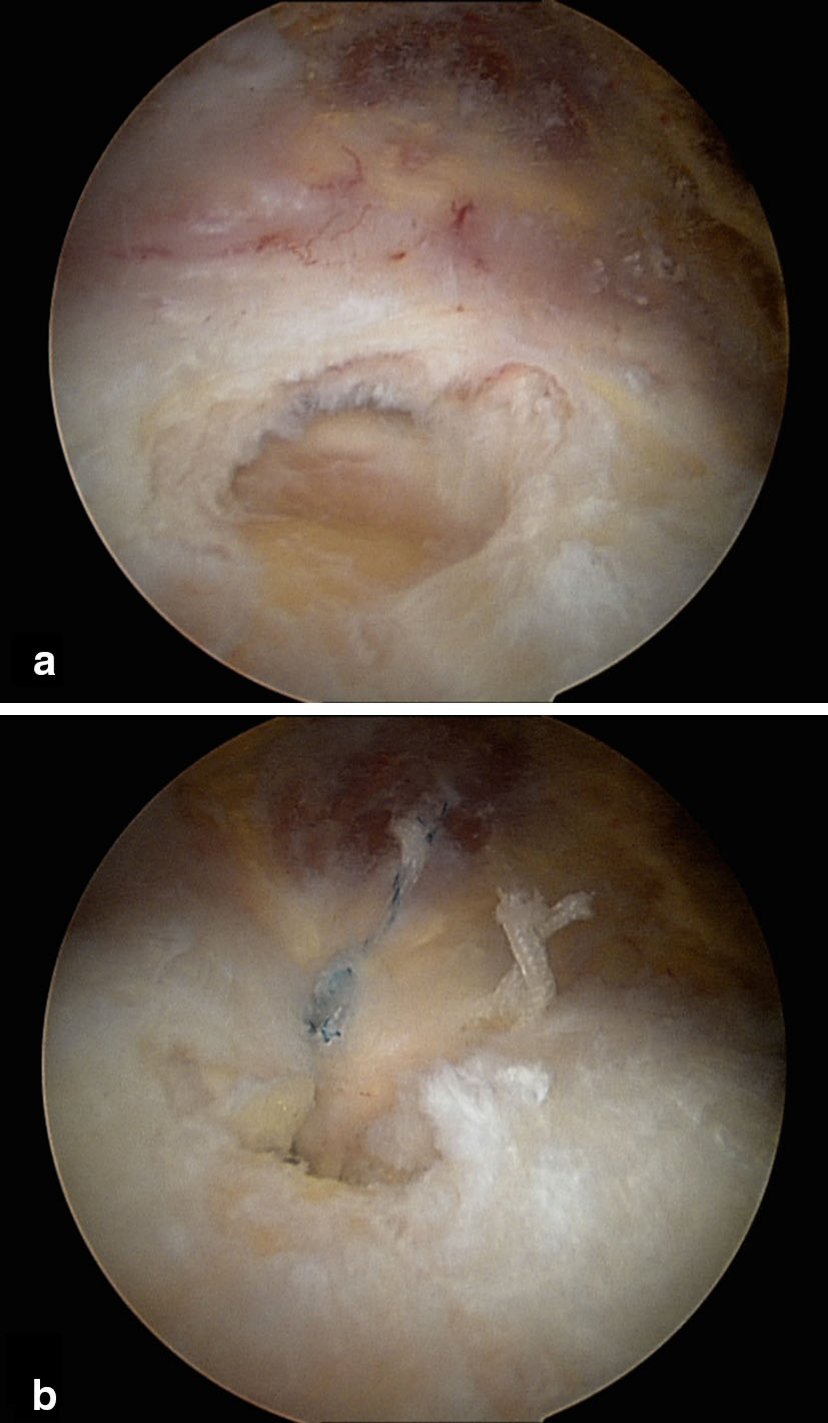
(**a**) Arthroscopic findings shows a complete insertional supraspinatus tendon tear after complete removal of a calcium deposit; (**b**) supraspinatus tendon-to-bone repair with a double suture anchor at the end of the arthroscopic procedure

## Aetiopathogenesis and histopathology

The aetiopathogenesis of CT remains elusive. Codman [2] hypothesised that overuse degeneration of rotator cuff leads to calcific deposits in the tendon, and this was also supported by Bishop [12], whereas Sandstrom [13] proposed that the degeneration in the tendon follows local ischaemia which led to calcium deposition. More recently, Mohr and Bilger [14] considered that the process begins with necrosis of tenocytes due to apoptosis along with intracellular accumulation of calcium, but a more detailed description was given by Uhthoff et al. [15], who proposed that the disease goes through three stages: precalcific, calcific and postcalcific. In the precalcific stage, there is fibrocartilaginous metaplasia in the tendon; this stage is rarely symptomatic. This is followed by the calcific stage, which is further divided into formative, resting and reabsorption phases. It is in the reabsorptive phase that patients are mostly symptomatic. The postcalcific phase is the healing phase, in which there is reabsorption of the deposit. Rui et al. [16] postulated that incorrect differentiation of stem cells, tendon-derived stem cells (TDSCs), into osteoblasts or chondrocytes could be the basis of the calcification. Disorders of the thyroid (thyroxine) or oestrogen metabolism may be related to the onset of the disease. Harvie et al. [17] reported endocrine involvement in 64.7 % of cases in their series, whereas Mavrikakis et al. [18] reported CT incidence in 31.8 % of their diabetic patients, compared with 10.3 % of the control group. Sengar et al. [19] found an increased frequency of human leucocyte antigen serotype class A1 in patients with CT. Mutation in the human homologue of the murine progressive ankylosis gene (*ANKH*) has been reported in patients with hereditary chondrocalcinosis, leading to alteration of the picture of extracellular inorganic pyrophosphate [20]. Oliva et al. [21] found that significantly increased expression of tissue transglutaminase (tTG)2 and its substrate osteopontin was detected in calcific areas compared with levels observed in normal tissue from the same subject with calcific tendinopathy. They concluded that a variation in the expression of these genes could be characteristic of this form of tendinopathy. The correlation between increased incidence of endocrine disorders and risk of developing CT remains unclear; similarly, the associations with genetic mutations, specific antigen serotypes and expression of tissue proteins need to be understood more deeply. One may speculate that patients with the aforementioned predisposing conditions may be at greater risk of developing CT. Furthermore, in this subpopulation of subjects, abnormal pre-existing calcifications can produce or enhance a complete RC tear, requiring a surgical approach.

## Imaging

### Conventional radiology

Standard radiographs in anterior–posterior (AP), outlet and axillary views are used for diagnosis and follow-up of CT, because they allow localisation and assessment of the texture and morphology of the deposits [22, 23] (Fig. 1). Many authors have tried to classify the deposits in terms of size [1] or morphology [6, 24–26] (Table 1). However, the fact that there are numerous classifications indicates that no classification perfectly correlates with the radiological picture and symptomatology of the patient, and there is also significant inter-observer variability [27].

**Table 1:** Radiographic classification of calcifying tendinitis of the shoulder

Author	Subtype	Description
Bosworth [1]	Small	<0.5 cm
	Medium	0.5–1.5 cm
	Large	1.5 cm
DePalma et al. [7]	Type I	Fluffy, amorphous and ill defined
	Type II	Defined and homogeneous
Molè et al. (French Arthroscopy Association) [27]	Type A	Dense, rounded, sharply delineated
	Type B	Multilobular, radiodense, sharp
	Type C	Radiolucent, heterogeneous, irregular outline
	Type D	Dystrophic calcific deposit
Gartner et al. [28, 29]	Type I	Well demarcated, dense
	Type II	Soft contour/dense or sharp/transparent
	Type III	Soft contour/translucent and cloudy

The location of the deposits in the tendons also varies [1, 22] (Table 2).

**Table 2:** Percentage of rotator cuff tendon involvement in calcifying tendinitis of the shoulder

Tendon	Percentage (%)
Supraspinatus	51
Infraspinatus	44.5
Teres minor	23.3
Subscapularis	3

### Ultrasound

Ultrasound (US) examination is a fundamental tool in diagnosis and treatment of CT [28, 29]. US has changed from having a purely diagnostic role to become an important therapeutic tool, especially for carrying out bursal lavage and tendon needling (Fig. 1b, c). Use of high-resolution US shows the presence of deposits and also defines their locations in the tendon, plus their size and texture. This technique shows RC tears in detail, and also enables staging of the deposits by correlation of shadow cones [30, 31]. In the resting phase, the deposits appear hyperechoic and arc shaped, whereas they appear non-arc shaped (fragmented/punctate, cystic, nodular) in the resolving phase [30]. These appearances can also be correlated with the symptomatic and asymptomatic phases of the disease [32]. Farin et al. [33] divided the deposits into three types: (1) hyperechoic focus with a well-defined shadow, (2) hyperechoic focus with a faint shadow and (3) hyperechoic focus with no shadow. Doppler examination during the nodular or cystic phase shows increased vascularity around the deposits [34], which correlates well with the histopathological findings of Uhthoff et al. [35], who showed how, during the reabsorption phase, the deposits are surrounded by phagocytes and there was concomitant proliferation of vascular channels around the deposits.

### Magnetic resonance imaging

Magnetic resonance imaging (MRI) is an additional but not essential imaging tool, because it does not give any additional information in most cases [36, 37]. Calcific deposits have low signal intensity in all MRI sequences, although areas of increased signal intensity can be found around deposits in T2 images, signifying oedema around the deposits in the resorptive phase. Such areas of increased signal intensity can be misinterpreted as a RC lesion [38, 39]. The accuracy of MRI in identifying calcific deposits is around 95 %, but it is more useful in cases of chronic CT, which may be associated with RC tears, AC and TO [10, 38, 40, 41] (Fig 2). All these investigations and a thorough clinical examination are of critical importance, especially when the primary disease is associated with signs and symptoms of other conditions, e.g., the stiffness occurring in the acute stage of the disease, which should be differentiated from that occurring in AC or secondary stiffness occurring in RC tears. Imaging must be used to differentiate chronic forms associated with TO from that occurring in association with dystrophic calcification or in tumours [42].

## Treatment options

Conservative management is always the first line of treatment. This includes non-steroidal anti-inflammatory drugs (NSAIDs), physiotherapy, UGN and ESWT. The outcome of conservative treatment was principally studied by Ogon et al. [43], who described prognostic factors whose identification was helpful for tailoring treatment for favourable outcome in the shortest possible time. They defined failure of nonoperative therapy as persistence of symptomatic calcific tendinitis of the shoulder after a minimum of 6 months of nonoperative treatment, including a minimum of 3 months of standardised nonoperative treatment. They concluded that the prognostic factors that significantly increased the probability of failure of nonoperative therapy (negative prognostic factors) were bilateral calcific deposit occurrence, localization near the anterior portion of the acromion, medial (subacromial) extension and high volume of calcific deposit. Prognostic factors that significantly reduced the probability of failure of nonoperative therapy (positive prognostic factors) were Gartner type III calcific deposit and lack of sonographic sound extinction of the calcific deposit. Treatment can be modulated depending upon the presence of these prognostic factors. Usually, the acute phase requires NSAIDs to relieve the pain and appropriate physiotherapy [passive range-of-motion (ROM) exercises] to avoid stiffness of the shoulder. Local steroid injection in the acute phase is a debatable topic, as studies have shown it to have positive [35] or no effect [44], or even a negative effect in the form of stopping reabsorption of the deposits [45]. In most cases, conservative treatment is sufficient for resolution of symptoms. Cho et al. [46] reported excellent to good results in 72 % of their patients.

### Ultrasound-guided needling

Although UGN was first demonstrated under fluoroscopy control by Comfort et al. [47], it was Farin et al. [33] who described use of US for bursal lavage and needling. Since then, it has been a commonly used intervention, as it is inexpensive and can be carried out on an out-patient basis under local anaesthesia (Fig. 1c). Gonzalez et al. [48] recently published a study of 121 patients with 2-year follow-up, reporting satisfactory results after UGN at 3 months. de Witte et al. [49] carried out a randomised controlled trial (RCT) between UGN with subacromial injection and subacromial injection alone; both groups showed improvement, but the UGN group fared better as compared with injection alone. A recent systemic review of literature [50] for the efficacy of UGN in CT concluded that, due to the variation in studies and the low quality of evidence, the efficacy of UGN could not be firmly established and additional high-quality studies are required.

### Extracorporeal shock wave therapy

ESWT has been used for medical treatment since the 1990s. Its use for CT is increasing, and like UGN, there is a lot of disparity, regarding the dosage (energy flux density), duration (impulses) and interval of administration of ESWT.

Low-energy (below 0.08 mJ/mm^2^), medium-energy (0.08–0.28 mJ/mm^2^) and high-energy (0.28–0.60 mJ/mm^2^) shock waves have been defined [51]. The shock waves can be generated through electrohydraulic, electromagnetic or piezoelectric mechanisms. Farr et al. [52] compared one dose of 0.3 mJ/mm^2^ versus two doses of 0.2 mJ/mm^2^, finding the former to be more effective. Ioppolo et al. [53] also published a RCT and found 0.20 mJ/mm^2^ dosage to be more effective than 0.10 mJ/mm^2^. Albert et al. [54] also found in favour of high-dose therapy, though their follow-up was only 3 months and they did not find any significant differences in the size of deposits on X-ray examination.

Various energy doses of ESWT have been reported for treatment of CT; most authors described good clinical outcomes with low- and medium-energy waves [51–53, 55–57]. The authors of a RCT [55] in which the control group was given sham treatment opined that the results were better in the ESWT group. The researchers also suggested other forms of treatment for patients who did not respond to ESWT after 6 months. Krasny et al. [56] compared ESWT alone and ESWT combined with UGN, finding that the combined treatment was more effective in relieving symptoms and that fewer patients in the combined treatment group required surgery. Daecke et al. [57] published long-term follow-up of patients managed with ESWT; although 20 % of all patients required surgery, 70 % of patients were treated successfully and no long-term complications were seen. Lee et al. [58] carried out a systematic review to determine the midterm effectiveness of ESWT, but due to the variability of treatment and reliability of the available studies, they were not able to come to a conclusion regarding a particular dosage of treatment. Kim et al. [59] carried out a comparative study between UGN and ESWT, finding better radiological and clinical outcomes in the UGN group, though both groups showed improvement relative to initial findings.

### Surgical treatment

After failure of conservative treatment modalities, surgical removal of the deposits is the remaining option. Although favourable results have been described with open removal of calcific deposits [4, 60–62], arthroscopy has become the preferred technique to treat the chronic formative phase of CT, offering results similar to open surgery but with less morbidity of the deltoid [63–69] (Fig. 3a, b). However, many issues remain under debate, such as repairing versus leaving the defect created, complete versus incomplete removal of the deposits and removal of deposits versus only acromioplasty. Ark et al. [64] published a report of 23 patients suggesting that complete removal of the deposits is not essential; they also did not attempt repair of the defects created following the removal of deposits. Other researchers [65, 66] have also made similar suggestions. In their study, Jerosch et al. [67] concluded that repair is not required following removal of the deposits, but they insisted on complete removal of the deposits. In contrast, Porcellini et al. [68] recommended complete removal of deposits followed by repair of the defect in the tendon, using simple side-to-side sutures or suture anchors depending upon the size of the residual defect. They argued that repair gives similar results without the fear of propagation of the tear and also helps in early patient rehabilitation. Tillander et al. [69] compared the outcome of acromioplasty in 50 patients: 25 with CT and another 25 with other causes of impingement syndrome. They did not find any significant difference between the Constant scores of the two groups at 2 years and recommended that the deposits should be left alone. However, other authors [64–68] recommended acromioplasty only in cases of visible mechanical impingement during arthroscopy, characterised by roughening of the ligament and osteophytes on the undersurface of the ligaments, as it did not have any additional benefit and the number of cases requiring acromioplasty varied in each of the studies.

Most authors [64–66, 68] recommended informing the patient about delayed recovery post-surgery and were of the opinion that surgical treatment should be reserved for patients not responding to conservative treatment for more than 6 months.

## Complications

In a recent review, Merolla et al. [11] described various complications associated with CT. They categorised pain as a complication, as the majority of patients with CT are asymptomatic. Other complications in their study were secondary AC and RC tears, both of which could occur during the primary disease or post-surgical intervention. They also pointed out ossifying tendinitis, which is an extremely rare condition occurring following surgical removal of calcium deposits. Many authors [10, 11, 38, 40, 41] have described TO of the greater tuberosity as an occurrence along with CT of the RC. Porcellini et al. [10] suggested that TO should be identified as a different form of CT which is prone to delayed recovery of patients managed conservatively and surgically. During UGN, mild vasovagal syncope may occur. High-dose ESWT is associated with pain sometimes requiring local anaesthesia, and local haematoma, erythema and ecchymosis have also been reported. Osteonecrosis of the humeral head has also been described [70].

## Overview

CT of the RC is a controversial topic with several treatment options that depend on the biologic stage of the disease. Although reabsorption occurs spontaneously in the majority of cases, a subpopulation of patients with persistent painful shoulder require conservative or operative management. In addition, some complications such as TO, AC or ossifying tendinitis (very rare) may give rise to prolonged pain resistant to common conservative therapies. UGN is indicated in the acute phase, but good results have also been found in patients with chronic calcific deposits. ESWT can be reasonably used in chronic calcific cases, even in combination with UGN. Surgical treatment should be considered when conservative measures have failed or in cases with US or MRI evidence of RC tears.
